# On the Mode in Which Chloroform Causes Death

**Published:** 1853

**Authors:** R. E. Bickersteth

**Affiliations:** Liverpool


*On the Mode in which Chloroform causes Death.*—By R. E. Bickersteth, Esq., of Liverpool.

From a number of experiments on the lower animals, confirmed by observations upon the manner in which chloroform acts upon man, Mr. Bickersteth arrives at the following conclusions:

1st. That in death from the inhalation of chloroform, the respiratory movements cease before the cardiac.

2d. That the heart continues its action, uninfluenced by the chloroform, for a period longer or shorter after the cessation of respiration, and that its then failing may be considered as a natural consequence of the respiration having ceased, and as independent of the influence of chloroform.

3d. That if, after the respiration has ceased, and while the heart is still in action, chloroform continues to be absorbed into the system, its movements may become impaired or cease—the chloroform in such case acting directly upon the heart.

4th. That if artificial respiration be resorted to before the cardiac contractions are seriously affected, and be properly maintained for a sufficient period, the respiratory function may be re-established.

The caution to be observed in administering chloroform, according to Mr. Bickersteth, is the same as that already inculcated by Mr. Clendon, viz., to watch the respiration, and not the pulse, and to cease the moment this begins to be disturbed. In cases of danger, the principal remedy is artificial respiration, and apropos of this topic are the following very excellent remarks:

"I would here direct attention to the expediency of drawing forward the tongue, in all cases when it is found necessary to resort to artificial respiration. When the patient is lying on the back, so soon as the breathing ceases and the jaw drops, the tongue is particularly liable to fall backwards and close the orifice of the glottis. Artificial respiration, under such circumstances, is worse than useless. It is better at once to pull the tongue well out of the mouth, and passing a hook through the tip, confide it to the care of an assistant. I am convinced that in some cases in which artificial respiration has failed, it has been from the neglect or too tardy adoption of this very simple means. Time of the utmost value has been lost in the absurd attempt to restore animation, by applying stimulants to the nostrils, or pouring cordials into the mouth, without even a thought that the first can have little or no effect after the respiration has ceased, or that the second would as likely pass into the trachea and bronchi as into the stomach."

*Edinburgh Monthly Journal*, Sept. 1853.

